# Trophoblasts Modulate the Ca^2+^ Oscillation and Contraction of Myometrial Smooth Muscle Cells by Small Extracellular Vesicle- (sEV-) Mediated Exporting of miR-25-3p during Premature Labor

**DOI:** 10.1155/2021/8140667

**Published:** 2021-08-07

**Authors:** Lin Wang, Wenzhu Zhang, Ning Zou, Lijuan Zhang

**Affiliations:** ^1^Department of Emergency Medicine, Shengjing Hospital of China Medical University, Shenyang, Liaoning, China; ^2^Department of Obstetrics and Gynecology, Shengjing Hospital of China Medical University, Shenyang, Liaoning, China

## Abstract

The placenta could transmit information to the maternal circulation via the secretion of small extracellular vesicles (sEVs), but little is known about whether and how these sEVs participate in premature labor (PTL). We speculate that miRNA plays an important role in sEV-mediated fetal-maternal information transmission. The present study was aimed at investigating whether the placenta can regulate the contraction of the maternal myometrium via sEV-mediated transmit of miR-25-3p during PTL. The expression of miR-25-3p and its targets Cav3.2 and SERCA2a in clinical samples, cells, and animal specimens was detected. The role of miR-25-3p was observed in the LPS-induced preterm labor mouse model. The sEVs from HTR-8/SVneo cells were characterized by transmission electron microscopy and nanoparticle tracking analysis. The Ca^2+^ oscillation in HMSMs was analyzed by an intracellular Ca^2+^ change tracking assay on a confocal microscope. The contraction of HMSMs was detected by a collagen matrix contraction assay. We found that miR-25-3p can directly bind to the 3′UTR of Cav3.2 and SERCA2a. The miR-25-3p level was decreased, and the expression of its targets Cav3.2 and SERCA2a was increased in the placenta and myometrium tissues of PTL patients. Forced upregulation of miR-25-3p reduced the oxidative stress and inflammation responses and the incidence of PTL in LPS-treated mice. The expression of miR-25-3p was not changed in LPS-stimulated human myometrial smooth muscle cells (HMSMs) but was strongly reduced in the trophoblast cell and its sEVs. Additionally, the trophoblast cell line HTR-8/SVneo could transmit miR-25-3p into HMSMs via sEVs. The sEVs derived from LPS-stimulated trophoblasts upregulated the expression of Cav3.2 and SERCA2a and triggered Ca^2+^ oscillation as well as the contraction of HMSMs; these effects were partially reversed by the overexpression of miR-25-3p in the trophoblasts. Further, the upregulation of miR-25-3p induced changes of Ca^2+^ oscillation and contraction of HMSMs mediated by sEVs which were also abrogated by the knockdown of miR-25-3p in HMSMs. The results demonstrated that miR-25-3p plays a crucial role in PTL via Cav3.2- and SERCA2a-mediated Ca^2+^ oscillation and contraction of HMSMs. PTL seems to be related to the decreased exosomal miR-25-3p content transmitted by the trophoblasts under inflammatory conditions.

## 1. Background

Births before 37 weeks of gestation are considered premature labor (PTL), which is known as an adverse pregnancy outcome [1]. PTL always has an increased risk of low birth weight, immaturity of multiple organs, serious chronic disabilities (such as respiratory distress syndrome, sepsis, and neurological disorders), or neonatal mortality that affects about 11% of all human newborns [2, 3]. The causes of PTL are complex and varied; oxidative stress, toxin stimulation, placental hypoxia, and immune or endocrine imbalance are all associated with PTL [4]. Also, infection of the placenta or embryolemma is believed to be the major cause of PTL [5]. The bacterial infection triggers an inflammatory response in the fetal membranes, myometrium, and cervix wall; then, the leucocytes accumulated and drive the progression to birth ahead of time [6]. However, the cascade from infection to labor onset and the complex molecular events implicated in this process remain to be fully elucidated.

The normal process of parturition is carried out according to a strict time course that is controlled by fetal phylogenetic clocks. However, there are still many doubts about how the fetus transmits signals to the maternal compartments. The 50-150 nm small vesicles containing miRNA, complex RNA, and proteins were now named small extracellular vesicles (sEVs), which were found to be important mediators in intercellular communication. Recently, the role of sEVs in information transmission during pregnancy has gradually attracted attention [7, 8]. The placenta releases sEV cargo fetal signals into the maternal circulation system as early as 6 weeks of gestation [8, 9]. More interestingly, the placental trophoblast could export miRNAs into maternal circulation via sEVs; then, these miRNAs were transferred to target tissues [8, 10], such as the uterus and cervix. Studies have identified different miRNA profiles in the sEVs of trophoblast cells from PTL [11, 12]. miRNAs are endogenous noncoding RNAs that participate in a variety of cellular biological processes by regulating their downstream target genes [13]. The changes in these placenta-derived miRNAs may be the causal factors in myometrial contractions.

Ca^2+^ is a fundamental second messenger in myometrial smooth muscle cells. The increased concentration of intracellular free Ca^2+^ is the most important factor controlling myometrial contractions [14]. The intracellular Ca^2+^ mainly comes from two sources, released by the sarcoplasmic reticulum or entering through the voltage-gated channels. Studies have suggested that the sarcoplasmic reticulum Ca^2+^-ATPase (SERCA) is involved in the depolarization, Ca^2+^ flow, and mechanical contraction of the myometrium [15, 16]; however, its role in PTL has not been fully reported. Voltage-gated channels can be classified into high-voltage-activated calcium channels (HVAs) and low-voltage-activated calcium channels (LVAs) due to their activation electrophysiological characteristics [17]. Among HVAs, only L-type Ca^2+^ channels are expressed in the myometrium. The role of L-type Ca^2+^ channels in uterine contraction and delivery has been widely investigated, and the L-type Ca^2+^ channel blocker nifedipine has been clinically applied to the treatment of PTL [18, 19]. The represented LVAs in the uterus are T-type calcium channels, which consist of three family members: Cav3.1 (*CACNA1G*), Cav3.2 (*CACNA1H*), and Cav3.3 (*CACNQ1I*). Recent studies have found that the T-type calcium channels also contribute to the Ca^2+^ influx in uterine smooth muscle cells and are closely related to uterine contraction [20–22]. In particular, the Cav3.2 protein was found to be upregulated in the uterine smooth muscle tissues of women experiencing preterm labor [23], although there is still limited information on the reason for the change.

Studies have suggested that miR-25-3p is involved in cardiac contraction by targeting SERCA2a [24, 25] and affects the mitochondrial Ca^2+^ uniporter in pulmonary artery smooth muscle cells [26]; however, its effects in PTL have not been explored. The present study found that miR-25-3p plays a pivotal role in Ca^2+^ oscillation and contraction of human myometrial smooth muscle cells (HMSMs) by the regulation of SERCA2a and Cav3.2. Further, we preliminarily revealed the process of PTL: infection changes the composition of a specific miRNA in the trophoblast sEVs (notably miR-25-3p), which twisted the signal transmitted to maternal HMSMs, thus affected the expression of Cav3.2 and SERCA2a, stimulated Ca2^+^ oscillation, triggered HMSM contraction, and resulted in PTL.

## 2. Materials and Methods

### 2.1. Tissue Specimens

The human myometrium and placenta were collected from patients who were hospitalized and cesarean delivered in Shengjing Hospital of China Medical University from Dec. 2018 to Dec. 2019. Five patients whose spontaneous labor was initiated at 28-37 gestational weeks which histopathologically identified bacterial infection were recruited into the preterm labor (PTL) group, and five patients over 37 gestational weeks that were not in labor were recruited into the full-term-not-in-labor (FNL) group. The spontaneous labor criteria were as follows: regular uterine contractions (<3 min apart) and cervical dilation that is more than 2 cm. Exclusion criteria were as follows: pregnant women with hypertension, diabetes, placental abruption, and placenta previa with heart disease, diabetes, and hypertension. The study was approved by the Medical Ethics Committee of the Shengjing Hospital of China Medical University (license number 2019PS072K).

### 2.2. LPS-Induced PTL in Mice

The animal study was approved by the Animal Research and Ethics of the Shengjing Hospital of China Medical University (license number 2015PS52K) and carried out the guidelines of the Research Center Ethics Committee for Animal Experimentation. The 8-10-week old C57BL/6 mice were purchased from Liaoning Shang Sheng Biotechnology Co. Ltd. (Benxi, China) with the license no. SCXK(Liao)2015-0001. The female and male mice were caged together, and the day when the vaginal suppository was found was recorded as pregnancy day 1 (P1). The pregnant mice were randomly divided into 5 groups (10 valid mice in each group). Mice in the LPS treatment group received 20 *μ*g LPS by intraperitoneal injection on P16 to mimic intrauterine infection [27, 28]. Mice in the LPS+NC agomir or LPS+miR-25-3p agomir group received 40 *μ*g/g NC agomir or miR-25-3p agomir by intraperitoneal injection on the day before LPS injection. Mice in the LPS+NNC55-0396 group were intraperitoneally injected with 40 *μ*g NNC55-0396 after LPS, 4 times a day until delivery. Parturition events were monitored at 7:00, 13:00, and 20:00 every day from P16 to P21, and the delivery that occurred before P19 was considered to be PTL. The mice were euthanized after delivery, and the uterine tissues were harvested and rapidly frozen in liquid nitrogen.

### 2.3. Measurement of Oxidative Stress and Inflammation Parameters

The placenta tissues were homogenized by mechanical homogenization, and the protein concentration was measured by using the BCA determination kit (Beyotime, Haimen, China). The SOD activity and MDA and GSH levels were detected by using commercial kits (Nanjing Jiancheng Bioengineering Institute, Nanjing, China) following the instructions. The TNF-*α* and IL-6 levels were measured by the enzyme-linked immunosorbent assay (ELISA, Multisciences, Hangzhou, China) as per the user's manual.

### 2.4. Real-Time PCR

The total RNA was extracted by using a total RNA extraction kit (Tiangen, Beijing, China). The purity of RNA was determined by using the OD260/OD280 ratio, and the RNA was available for subsequent study when the ratio was between 1.8 and 2.0. 0.4 *μ*g/ml RNA was added to the reverse transcriptional system, and the reverse transcription was performed by using the M-MLV reverse transcription kit (Tiangen). The reverse transcription reaction condition is as follows: 25°C for 10 min, 42°C for 50 min, and 80°C for 10 min. The real-time PCR reaction system is as follows: 1 *μ*l cDNA, forward and reverse primer 0.5 *μ*l each, 0.3 *μ*l SYBR Green (Solarbio), and 10 *μ*l 2× Taq PCR Master Mix (Tiangen), making up to 20 *μ*l with ddH_2_O. The primer sequences used in the experiment are listed in Supplementary Table 1. The PCR reactions were carried out on Exicycler™ 96 (Bioneer, Daejeon, Korea), and the reaction condition is as follows: 94°C for 5 min; 94°C for 10 s, 60°C for 20 s, 72°C for 30 s, and 40 cycles; 72°C for 2.5 min; 40°C for 1.5 min; and melting from 60°C to 94°C; the products were incubated at 25°C for 1-2 min.

### 2.5. Immunoblotting Assay

The proteins were extracted by using the RIPA lysate solution (Solarbio, Beijing, China); then, the equivalent amount of protein was separated by 5%, 8%, or 15% SDS-PAGE. After being transferred onto the PVDF membranes, the blots were blocked with fat-free milk for 1 hour. The membranes were then probed with primary antibodies such as CD63 antibody (1 : 1000 dilution, 25682-1-AP, Proteintech, Wuhan, China), CD81 antibody (1 : 2000 dilution, ab109201, Abcam, Cambridge, MA, USA), Cav3.2 antibody (1 : 500 dilution, 28358-1-AP, Proteintech), SERCA2 antibody (1 : 1000 dilution, DF6240, Affinity Biosciences, Zhenjiang, China), or GAPDH antibody (1 : 10000 dilution, 60004-1-Ig, Proteintech). The specific band was then detected by using a horseradish peroxidase- (HRP-) conjugated goat anti-rabbit antibody (1 : 3000 dilution, SE134, Solarbio) or goat anti-mouse-antibody (1 : 3000 dilution, SE131, Solarbio) followed by enhanced chemiluminescence (Solarbio).

### 2.6. Immunohistochemistry

The myometrium or placenta tissues were fixed in 4% paraformaldehyde, embedded in paraffin, cut into 5 *μ*m slices, dewaxed in xylene, and then dehydrated in graded ethanol. The slices were blocked with normal goat serum following incubation with the primary antibodies against Cav3.2 (1 : 50 dilution, sc-377510, Santa Cruz, CA, USA), SERCA2a (1 : 50 dilution, ab150435, Abcam, Cambridge, MA, USA), or 4-hydroxynonenal (4-HNE, 1 : 100 dilution, MAB3249, Novus Biologicals, Littleton, Colorado, USA) at 4°C overnight. The slices were subsequently rinsed in PBS and incubated with a HRP-conjugated goat anti-rabbit secondary antibody (1 : 500 dilution, #31460, Thermo Fisher Scientific, Waltham, MA, USA) or HRP-conjugated goat anti-mouse secondary antibody (1 : 100 dilution, #31430, Thermo Fisher Scientific, Waltham, MA, USA) at 37°C for 60 min. After being rendered by the DAB reagent, the slices were counterstained with hematoxylin and examined under the BX53 microscope system (Olympus, Tokyo, Japan).

### 2.7. Cell Culture and Treatment

Primary human myometrial smooth muscle cells (HMSMs) were isolated as previously described [29, 30] and then identified by immunofluorescence detection of *α*-SMA. The cells were cultured in DMEM supplemented with 7.5% fetal calf serum, penicillin 40 U/ml, and streptomycin 40 *μ*g/ml. The medium was changed every 2-3 days, and the cells of the 3^rd^-6^th^ generation were employed for the following studies. The trophoblast cell line HTR-8/SVneo was generously provided by Dr. Charles H. Graham (Department of Anatomy & Cell Biology, Queen's University at Kingston, Canada) and cultured in RPMI-1640 medium supplemented with 5% fetal calf serum plus 1% penicillin/streptomycin. Cells in the LPS treatment group were stimulated with 1 *μ*g/ml LPS (Escherichia coli O55:B5, Sigma-Aldrich Int., Louis, MO, USA). To up- or downregulate the miR-25-3p, the cells were transfected with 50 nmol/l miR-25-3p agomir or antagomir (GenePharma, Shanghai, China) according to the instructions of the Lipofectamine RNAiMAX Transfection Agent (Invitrogen, Carlsbad, CA, USA).

### 2.8. sEV Isolation, Transmission Electron Microscopy (TEM), and Nanoparticle Tracking Analysis (NTA)

sEVs from HTR-8/SVneo cells were extracted by the ultracentrifugation method as previously reported [31, 32]. For transmission electron microscopy detection, the sEVs were fixed with 2% paraformaldehyde, loaded on copper grids, negatively stained with phosphotungstic acid, and then observed by using transmission electron microscopy (Hitachi-HT7700, 100 kV, Hitachi, Chiyoda, Japan). The average size and particle concentration were automatically monitored by the NanoSight NS300 instrument (Malvern Instruments, Westborough, MA, USA).

### 2.9. The Internalization of sEVs into HMSMs

The sEVs from HTR-8/SVneo cells were incubated with 1 *μ*g/ml Dil (MaoKangbio, Shanghai, China) at 37°C for 20 min, and the excess dyestuff was removed by centrifugation. Then, the 400 *μ*g/ml Dil-labeled sEVs were added to the HMSM culture medium and incubated for 24 h at 37°C. After washing, the HMSMs were fixed with 4% paraformaldehyde and counterstained with DAPI. The internalized sEVs were observed under the BX53 microscope system (Olympus, Tokyo, Japan).

### 2.10. The Delivery of miR-25-3p by sEVs

The delivery of miR-25-3p by sEVs was examined by the transwell coculture assay as previously reported [33]. In brief, the HTR-8/SVneo cells were pretransfected with Cy3-miR-25-3p mimic and seeded (0.1 × 10^6^/well) in the upper chamber that was precoated with a 0.4 *μ*m polycarbonated filter. The lower chamber was seeded with the same number of HMSMs. The HTR-8/SVneo cells incubated with Cy3 dye without miR-25-3p conjugation served as a control. The fluorescence was observed under the microscope, and the miR-25-3p level of the HMSMs was detected by real-time PCR 12 h after the coculture.

### 2.11. Dual-Luciferase Assay

**Figure 1:**
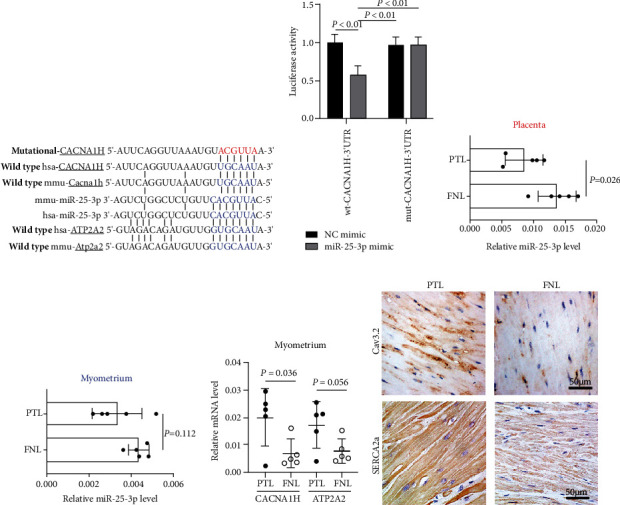
miR-25-3p targets *ATP2A2* and *CACNA1H*, and the expression of miR-25-3p was reduced in the infection-related PTL placenta. The binding sites of miR-25-3p on *ATP2A2* (the gene name of SERCA2a) and *CACNA1H* (the gene name of Cav3.2) (a). The binding activity of miR-25-3p on *CACNA1H* was measured by the dual-luciferase assay (b). The expression of miR-25-3p in the clinical placenta or myometrium tissue was determined by real-time PCR (c, d). The expression of *CACNA1H* and *ATP2A2* in clinical myometrium tissue was detected by real-time PCR (e). Immunohistochemistry detection of Cav3.2 and SERCA2a in the clinical myometrium tissues (f). PTL: preterm labor; FNL: full-term-not-in-labor.

The wild-type or mutated 3′-untranslated region (UTR) of *CACNA1H* was cloned into the pmirGLO luciferase reporter plasmid (Promega, Madison, WI, USA) and named wt-*CACNA1H*-3′UTR or mut-*CACNA1H*-3′UTR. The mutated sites are shown in Figure 1(a). The wt-*CACNA1H*-3′UTR or mut-*CACNA1H*-3′UTR was cotransfected into HEK293T cells with negative control (NC) mimic or miR-25-3p mimic. After 24 h, the luciferase activity was detected by using the dual-luciferase detection kit according to the manufacturer's instruction (KeyGEN, Nanjing, China).

### 2.12. Intracellular Ca^2+^ Change Tracking Assay

The intracellular Ca^2+^ change tracking assay was performed by Dalrymple et al. [34] described with little modification. Briefly, the HMSMs were exposed to 400 *μ*g/ml sEVs for 48 h. After that, the cells were loaded with 5 *μ*M Fura-3-AM (Beyotime, Haimen, China) and incubated at 37°C for 30 min. The cells were perfused with precooled physiological salt solution (PSS, NaCl 140 mM, KCl 5.9 mM, NaH_2_PO_4_ 1.2 mM, NaHCO_3_ 5 Mm, MgCl_2_ 1.4 mM, CaCl_2_ 1.8 mM, glucose 11.5 mM, and pH 7.4 HEPES 10 mM) for 200 s at the beginning of the test; then, the cells were imaged every 4-6 s, and the fluorescence intensity was measured under a confocal microscope (Nikon A1+, Nikon Instruments, Melville, NY). The test was performed at 3 treatments: (I) to evaluate the role of SERCA2a, the cells were stimulated with 5 *μ*M cyclopiazonic acid (CPA) to release the intracellularly stored Ca^2+^. (II) To investigate whether the exogenous Ca^2+^ was involved, the cells were perfused with Ca^2+^-free PSS for 200 s and then resumed with the physiological concentration of Ca^2+^ (1 mM). (III) The cells were pretreated with PSS for 200 s and then applied with 100 nM oxytocin (OXT) to stimulate extracellular Ca^2+^. After exposing cells to OXT for 200 s, 100 *μ*M NNC 55-0396 (the L-type Ca^2+^ channel antagonist) was added to determine the effect of Cav3.2.

### 2.13. Collagen Matrix Contraction Assay

The HMSM contraction was measured as previously described [35]. The 24-well plates were precoated with 2% bovine serum albumin. The HMSMs were exposed to 400 *μ*g/ml sEVs for 48 h and then resuspended with DMEM supplemented with 12% FBS and adjusted to 7.5 × 10^5^ cells/ml. The cells were then mixed with an equal volume of cell matrix type IA (Solarbio) and 2× DMEM solution and incubated at 37°C till gelation. After detaching, the contracted collagen gels were photographed.

### 2.14. Statistical Analysis

The data are expressed as mean ± SD. All data were in a normal distribution, which was tested by the Kolmogorov-Smirnov test (*P* > 0.1), and the outliers were excluded. The experiments in cells were repeated more than three times. The in vivo experiments include at least ten valid data in each group. The differences between the two groups were analyzed by Student's *t*-test; the differences among more than two groups were analyzed by one way-ANOVA. Data were analyzed by GraphPad Prism ver. 8.0 (San Diego, CA, USA). Changes were considered statistically different when *P* < 0.05.

## 3. Results

### 3.1. miR-25-3p Could Bind to the 3′UTR of the *CACNA1H* and *ATP2A2* mRNA

*CACNA1H* is the gene name of Cav3.2, and *ATP2A2* is the gene name of SERCA2a. The bioinformatics analysis suggested that both the murine and the human 3′UTR of the *CACNA1H* and *ATP2A2* contained the binding site of miR-25-3p (Figure 1(a)). The binding of miR-25-3p on *ATP2A2* has been proven by Wahlquist and colleagues [24]. Thus, the binding activity of miR-25-3p on the *CACNA1H* was measured by the dual-luciferase assay in the present study, as displayed in Figure 1(b), the luciferase activity of the cells transfected with wild-type 3′UTR and miR-25-3p mimic was significantly reduced, and these results identified that miR-25-3p directly binds to the 3′UTR of *CACNA1H*.

### 3.2. The Expression of miR-25-3p Was Reduced in the Infection-Related PTL Placenta

We further tested the miR-25-3p level in clinical samples, and the results exhibited that, when compared to the FNL group, the miR-25-3p level of the PTL group was significantly reduced in the placenta tissues (Figure 1(c)) and also decreased in the myometrium tissues, although the difference is not significant (Figure 1(d)). Meanwhile, an elevation of *CACNA1H* and *ATP2A2* was noted in the myometrium tissues from the PTL group (Figure 1(e)). The immunohistochemistry results also confirmed the higher levels of Cav3.2 and SERCA2a in the PTL myometrium (Figure 1(f)). These results indicate that miR-25-3p and its targets Cav3.2 and SERCA2a may play a role in the onset of labor.

### 3.3. Forced Upregulation of miR-25-3p Alleviated Oxidative Stress and Inflammation Response in the Placenta of LPS-Induced PTL Mice

**Figure 2:**
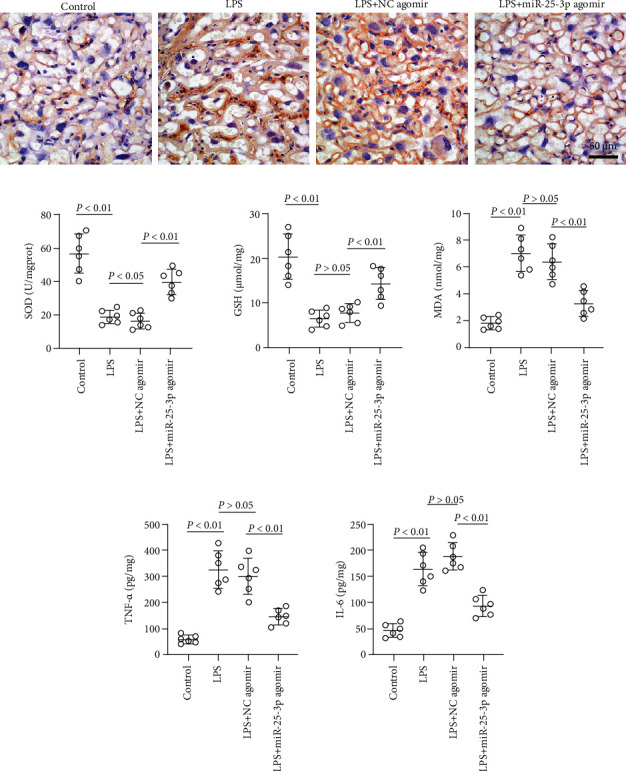
miR-25-3p alleviated LPS-induced oxidative stress and inflammation response in the placenta. The pregnant mice were injected with 20 *μ*g LPS at P16; then, the placenta tissues were collected at 7 h after LPS injection. The expression of 4-hydroxynonenal (4-HNE) in the placenta was detected by immunohistochemistry (a). The superoxide (SOD) activity and glutathione (GSH) and malondialdehyde (MDA) levels in the placenta were determined by using commercial kits (b). The TNF-*α* and IL-6 levels in the placenta were also detected (c).

Oxidative stress and inflammation response are involved in numerous birth outcomes, especially in PTL [36, 37]; thus, the effect of miR-25-3p on oxidative stress and inflammation response was determined. As shown in Figure 2(a), the expression of 4-hydroxynonenal (4-HNE), a marker of oxidative damage, was increased in the placenta of LPS-induced PTL mice and this was revised by the upregulation of miR-25-3p. Similarly, the SOD activity and the GSH and MDA levels that were changed by LPS treatment were all reversed by the upregulation of miR-25-3p (Figure 2(b)). At the same time, LPS-induced upregulation of TNF-*α* and IL-6 levels was also abrogated by miR-25-3p agomir (Figure 2(c)). These data suggested that miR-25-3p protected the placenta from LPS-induced oxidative stress and inflammation injuries.

### 3.4. Forced Upregulation of miR-25-3p Alleviated LPS-Induced PTL in Mice

**Figure 3:**
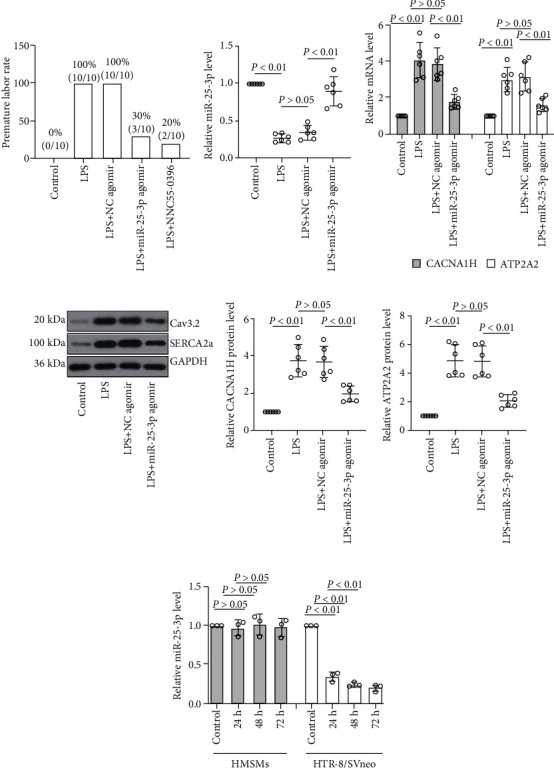
miR-25-3p alleviated LPS-induced premature labor (PTL) by targeting *ATP2A2* and *CACNA1H*. The pregnant mice were injected with 20 *μ*g LPS at P16, and the premature labor rate was calculated as PTL mice number/total mice number in each group (10 valid mice in each group) (a). The expression of miR-25-3p in the myometrium was examined by real-time PCR (b). The expression of *CACNA1H* and *ATP2A2* in the myometrium was examined by real-time PCR (c). The expression of Cav3.2 and SERCA2a in the myometrium was examined by western blot (d). The expression of miR-25-3p in LPS-stimulated HMSMs or HTR-8/SVneo was determined by real-time PCR (e). HMSMs: human myometrial smooth muscle cells.

To evaluate whether miR-25-3p is involved in the regulation of premature delivery, the LPS-induced PTL model was established. As shown in Figure 3(a), the PTL rate in the LPS group was 100% and decreased to 20% in the NNC 55-0396 (L-type Ca^2+^ channel antagonist) administration group, which was employed as the positive control. At the same time, the PTL rate was reduced to 30% by the upregulation of miR-25-3p. The expression of miR-25-3p in the myometrium was further detected, and the data showed that the expression of miR-25-3p was reduced in the myometrium that was stimulated with LPS and was upregulated by the treatment with miR-25-3p agomir (Figure 3(b)). As expected, the Cav3.2 and SERCA2a mRNA and protein levels were all increased when exposed to LPS and reduced by the treatment of miR-25-3p agomir (Figures 3(c) and 3(d)). These data indicate that miR-25-3p may ameliorate PTL, partly by the regulation of Cav3.2 and SERCA2a.

### 3.5. The Expression of miR-25-3p Was Reduced in LPS-Stimulated Trophoblasts

The pivotal roles of Cav3.2 and SERCA2a in regulating Ca^2+^ flow prompt us to examine whether the expression of miR-25-3p is correlated with the Ca^2+^ oscillation in the HMSMs. LPS can mimic the infected microenvironment of the myometrium [38, 39]; moreover, we have found that treatment with LPS increased the calcium concentration of uterine smooth muscle cells [40]. Thus, the HMSMs were stimulated with LPS; then, the expression of miR-25-3p was measured. Surprisingly, the miR-25-3p level was not changed by the treatment of LPS (Figure 3(e)). Considering that the maternal myometrium is predominantly associated with the placenta state during pregnancy, therefore, the trophoblasts, HTR-8/SVneo, were exposed to LPS and the expression of miR-25-3p was detected. As shown in Figure 3(e), the expression of miR-25-3p was decreased with the prolongation of stimulation time. Based on these results, we speculate that compared to HMSMS, the trophoblast cells may be more sensitive to the LPS.

### 3.6. Trophoblasts Transmit miR-25-3p to HMSMs by the Secretion of sEVs

**Figure 4:**
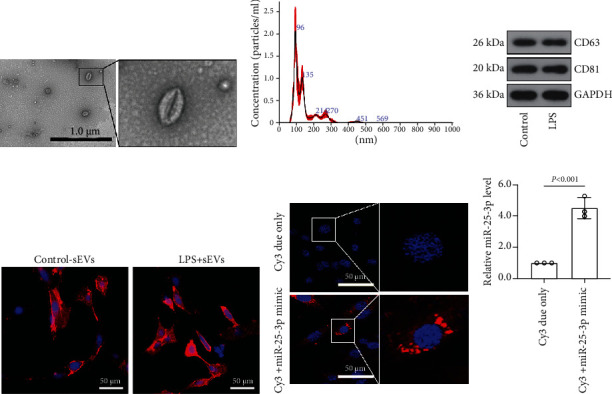
The transfer of exosomal miR-25-3p into HMSMs. The sEVs derived from HTR-8/SVneo cells were observed under transmission electron microscopy (TEM) (a). The sEVs were analyzed by Nanoparticle Tracking Analysis (NTA) (b). The sEV markers were detected by western blot (c). The HMSMs were incubated with Dil-labeled sEVs for 24 h, and the fluorescence was observed (d). Red: Dil, blue: DAPI. The HTR-8/SVneo cells were transfected with the Cy3-conjugated miR-25-3p mimic or Cy3 dye only. Then, the cells were cocultured with HMSMs for 12 h, and the fluorescence of HMSMs was detected (e). Red: Cy3, blue: DAPI. The expression of miR-25-3p in HMSMs was measured by real-time PCR (f). HMSMs: human myometrial smooth muscle cells.

The placenta is more sensitive to environmental stimulus and could transmit diverse signaling molecules to the matrix by the secretion of sEVs [8, 10, 41]. Therefore, we want to know whether the miR-25-3p was exported from the trophoblast to the myometrium via the sEVs. Herein, the sEVs from the placental trophoblastic cells HTR-8/SVneo were isolated, and the TEM results showed that the sEVs derived from HTR-8/SVneo exhibited the typical double-layer vesicle structure (Figure 4(a)). The Nanoparticle Tracking Analysis (NTA) showed that the size distribution of these particles was ranged from 30 to 200 nm (Figure 4(b)). Besides, these sEVs are enriched in the specific tetraspanin family proteins CD63 and CD81 (Figure 4(c)). These results confirmed that the sEVs were successfully extracted from the trophoblast cells.

Further, the HMSMs were incubated with Dil-labeled sEVs for 24 h, and a strong red fluorescence was observed by the fluorescence microscopy (Figure 4(d)), indicating that these sEVs were internalized by the HMSMs. To investigate whether these trophoblast-derived sEVs could transmit miRNAs to HMSMs, the HTR-8/SVneo cells were transfected with Cy3-conjugated miR-25-3p mimic or Cy3 dye only. Then, the cells were seeded in the upper chamber and cocultured with HMSMs in the lower chamber. As shown in Figure 4(e), the red fluorescent appearance in the HMSMs suggested that the Cy3-miR-25-3p mimic was delivered from the HTR-8/SVneo cells to the recipient HMSMs. Notably, the miR-25-3p level was increased concomitant with the red fluorescence (Figure 4(f)). Taken together, the trophoblast cells can transmit miR-25-3p to HMSMs via sEVs.

### 3.7. The sEVs from LPS-Stimulated Trophoblasts Induced Ca^2+^ Oscillation and Affect Contraction of HMSMs

**Figure 5:**
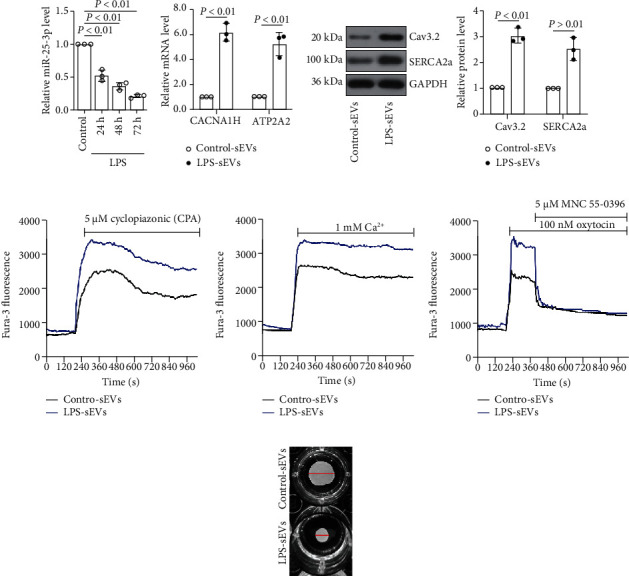
The sEVs from LPS-stimulated trophoblasts induced Ca^2+^ oscillation and affect contraction of HMSMs. The HTR-8/SVneo cells were stimulated with LPS for different time points; then, the sEVs were derived, and the expression of miR-25-3p was measured by real-time PCR (a). The HTR-8/SVneo cells were stimulated with LPS, and sEVs were isolated and applied to the HMSMs; the expression of *CACNA1H* and *ATP2A2* in the HMSMs was determined by real-time PCR (b). The expression of Cav3.2 and SERCA2a in HMSMs was measured by western blot (c). The sEVs were derived from the HTR-8/SVneo cells with or without the stimulation of LPS and then applied to the HMSMs. The Ca^2+^ transients of HMSMs were measured in the presence of cyclopiazonic acid (CPA) (d). The cells were preincubated with the indicated sEVs and perfused with Ca^2+^ free PSS and then resumed with Ca^2+^; next, the Ca^2+^ transients of HMSMs were measured (e). The cells were preincubated with the indicated sEVs and then stimulated with oxytocin for 200 s; after that, NNC 55-0396 (the L-type Ca^2+^ channel antagonist) was added, and the Ca^2+^ transients of HMSMs were measured (f). The HMSMs were preincubated with the indicated sEVs; then, the contraction was detected by the collagen matrix contraction assay (g).

To characterize the changes of the exosomal composition induced by LPS, the expression of miR-25-3p in the sEVs from HTR-8/SVneo was detected and we found that LPS treatment resulted in a dramatic reduction of exosomal miR-25-3p (Figure 5(a)). We further evaluated the expression of Cav3.2 and SERCA2a in HMSMs that were exposed to these sEVs. The data showed that stimulation with the sEVs from LPS-treated cells significantly increased the Cav3.2 and SERCA2a levels in HMSMs (Figures 5(b) and 5(c)). These findings revealed that the sEVs derived from LPS-treated HTR-8/SVneo cells promoted the expression of Cav3.2 and SERCA2a in HMSMs.

The Ca^2+^ change tracking assay was employed to evaluate the Ca^2+^ transients induced by these sEVs. As previously suggested [42], the CPA was applied to trigger the release of intracellularly stored Ca^2+^. A higher peak of Ca^2+^ response was found in the HMSMs treated with the sEVs derived from LPS-stimulated HTR-8/SVneo cells (Figure 5(d)), indicating that the sEVs from LPS-treated cells promoted the release of intracellularly stored Ca^2+^. The HMSMs were preincubated with Ca^2+^-free PSS; then, the readdition of Ca^2+^ resulted in a higher peak, demonstrating that the sEVs from LPS-treated cells also altered the Ca^2+^ entry (Figure 5(e)). Thus, oxytocin was applied to stimulate the extracellular Ca^2+^, as shown in Figure 5(f), the sEVs from LPS-treated HMSMs exhibited stronger oscillation, and these were blocked by the addition of NNC 55-0396 (the T-type Ca^2+^ channel antagonist), indicating that the sEVs from LPS-treated cells may also affect the Ca^2+^ entry via the T-type Ca^2+^ channel. The HMSM contraction was further detected, and the results suggested that the sEVs from LPS-stimulated cells also enhanced the contraction of HMSMs (Figure 5(g)). These results indicated that the sEVs from LPS-stimulated HTR-8/SVneo cells facilitated the contraction of HMSMs by the promotion of Ca^2+^ oscillation.

### 3.8. Overexpression of miR-25-3p Abrogated sEV-Induced Ca^2+^ Oscillation and HMSM Contraction

**Figure 6:**
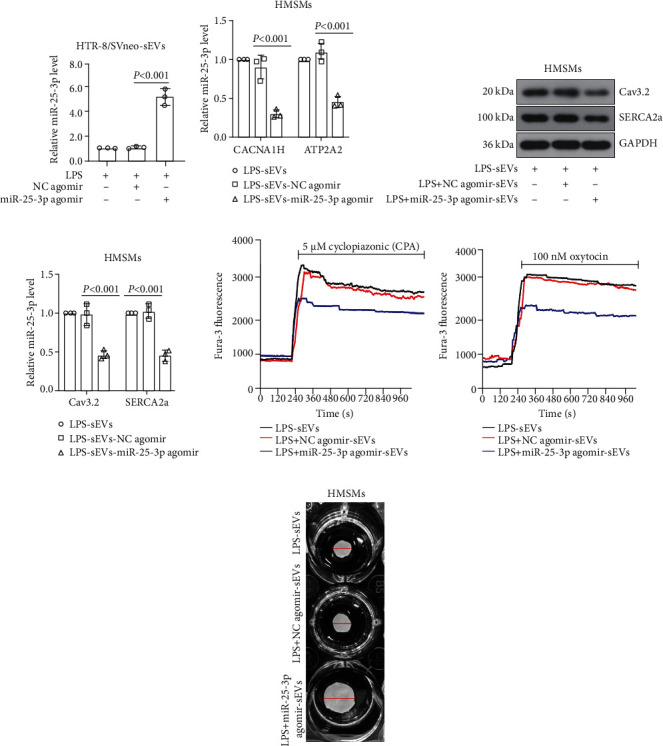
Overexpression of miR-25-3p abrogated sEV-induced Ca^2+^ oscillation and HMSM contraction. The HTR-8/SVneo cells were pretransfected with miR-25-3p agomir before treatment with LPS, and the expression of miR-25-3p in the sEVs was measured by real-time PCR (a). The HTR-8/SVneo cells were pretransfected with miR-25-3p agomir before treatment with LPS; then, the sEVs were derived and applied to HMSMs. The expression of *CACNA1H* and *ATP2A2* in the HMSMs was determined by real-time PCR (b). The expression of Cav3.2 and SERCA2a in HMSMs was measured by western blot (c), and the densitometry was analyzed (d). The cells were preincubated with the indicated sEVs; then, the Ca^2+^ transients of HMSMs were measured in the presence of cyclopiazonic acid (CPA) (e). The cells were preincubated with the indicated sEVs; then, the Ca^2+^ transients of HMSMs were measured at the presence of oxytocin (f). The cells were preincubated with the indicated sEVs; then, the contraction of HMSMs was detected by the collagen matrix contraction assay (g).

To delineate the effect of miR-25-3p in sEV-mediated Ca^2+^ oscillation, the HTR-8/SVneo cells were pretransfected with miR-25-3p agomir before treatment with LPS. The real-time PCR results showed that the miR-25-3p level in the sEVs was significantly increased by miR-25-3p agomir (Figure 6(a)). Then, the sEVs were collected and added to the HMSMs, as shown in Figure 6(b)–6(d); these miR-25-3p upregulated sEVs caused a decrease in the Cav3.2 and SERCA2a in the HMSMs. The Ca^2+^ change tracking assay showed that the LPS-induced intracellular Ca^2+^ release (Figure 6(e)) or extracellular Ca^2+^ entry (Figure 6(f)) was both reversed by the forced upregulation of miR-25-3p. Besides, the LPS-induced contraction of HMSMs was also abrogated by the miR-25-3p agomir (Figure 6(g)). These data indicated that the change of miR-25-3p in trophoblast cells affects the contraction of HMSMs via sEV-mediated Ca^2+^ responses.

### 3.9. The Inhibition of miR-25-3p in HMSMs Reversed Exosome-Induced Ca^2+^ Oscillation and Contraction

**Figure 7:**
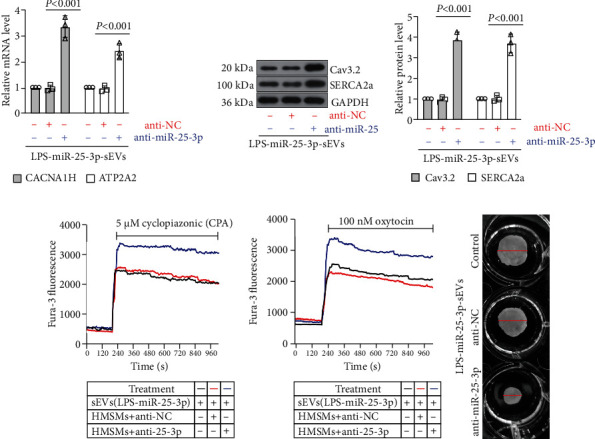
The inhibition of miR-25-3p in HMSMs reversed exosome-induced Ca^2+^ oscillation and contraction. The HMSMs were transfected with miR-25-3p antagomir before being applied with the sEVs derived from HTR-8/SVneo (the HTR-8/SVneo cells have been transfected with miR-25-3p agomir and treated with LPS), and the expression of *CACNA1H* and *ATP2A2* in the HMSMs was determined by real-time PCR (a). The expression of Cav3.2 and SERCA2a in HMSMs was measured by western blot (b). The cells were preincubated with the indicated sEVs; then, the Ca^2+^ transients of HMSMs were measured in the presence of cyclopiazonic acid (CPA) (c). The cells were preincubated with the indicated sEVs; then, the Ca^2+^ transients of HMSMs were measured in the presence of oxytocin (d). The cells were preincubated with the indicated sEVs; then, the contraction of HMSMs was detected by the collagen matrix contraction assay (e).

To further identify the contribution of miR-25-3p in HMSM contraction, the HMSMs were transfected with miR-25-3p antagomir before being applied with the sEVs derived from HTR-8/SVneo (the HTR-8/SVneo cells have been transfected with miR-25-3p agomir and treated with LPS). As shown in Figures 7(a) and 7(b), the sEV-induced downregulation of Cav3.2 and SECA2a in HMSMs was restored by the inhibition of miR-25-3p. The sEVs associated with intracellular Ca^2+^ release and extracellular Ca^2+^ entry in HMSMs were all reversed by the inhibition of miR-25-3p in HMSMs (Figures 7(c) and 7(d)). Following the changes of Ca^2+^, the suppressed cell contraction by the sEVs was also abrogated by the inhibition of miR-25-3p (Figure 7(e)). These data demonstrated that miR-25-3p is critical in regulating the HMSM contraction.

## 4. Discussion

The present study demonstrated that (I) when compared to the FNL tissues, the miR-25-3p level was decreased in the clinical placenta and myometrium in PTL tissues; meanwhile, its targets Cav3.2 and SERCA2a were increased in the myometrium. (II) Forced upregulation of miR-25-3p counteracted LPS-induced oxidative stress, inflammation reactions, and PTL and reduced Cav3.2 and SERCA2a levels in the myometrium of PTL mice. (III) The trophoblast cell was more sensitive to LPS when compared to the HMSMs, and the trophoblast cells can transmit miR-25-3p to HMSMs via sEVs; moreover, treatment with LPS reduced the exosomal miR-25-3p of trophoblast cells. (IV) The sEVs from LPS-stimulated trophoblast cells increased the Cav3.2 and SERCA2a levels in HMSMs and enhanced the Ca^2+^ oscillation and contraction of HMSMs. (V) These sEVs induced Ca^2+^ oscillation and contraction of HMSMs partly restored by the regulation of miR-25-3p. To our knowledge, this is the first study to reveal that the placenta affects maternal uterine contraction via exosomal miRNA in PTL.

At present, treatment for PTL is predominantly focused on suppressing the onset of labor; thus, it is recognized that effective therapies should target myometrial contractility. We first detected several cell contraction-associated miRNAs in the clinical PTL and FNL myometrium tissues, and we found that only miR-25-3p was changed (data not shown). Wahlquist et al. [24] have proven that miR-25-3p could inhibit the Ca^2+^ pump protein SERCA2a expression by binding to its 3′-UTR. We further identified that one of the T-type calcium channels, Cav3.2, is also a target of miR-25-3p. The Cav3.2 and SERCA2a level was elevated in the PTL myometrium along with the decreased miR-25-3p, proposing that miR-25-3p and its targets may contribute to the PTL. The colonization of Gram-negative germs is a pivotal risk factor for PTL [43, 44]. LPS can induce preterm labor by evoking the release of proinflammatory cytokines and is sometimes used to mimic infection-related PTL in murine [45, 46]. Environmental exposures that induce oxidative stress and inflammation often contribute to the risk of PTL. Elevated levels of oxidative stress markers were found in preterm neonates and mothers that negatively correlate with birth weight and gestational age at birth [47–49]. miR-25-3p has been reported to suppress oxidative stress and inflammation in several pathological processes [50, 51]. The LPS-induced PTL was strongly restrained by the overexpression of miR-25-3p in mice, and these results identified the pivotal role of miR-25-3p in PTL.

Previous studies have revealed that SERCA2 was increased in the myometrium during labor [52], and the upregulated SERCA2 activated the release of store-operated Ca^2+^ in the myometrium [15]. More importantly, the increased miR-25-3p level injured the contractile function of the heart muscle cells via the suppression of SERCA2a [24]. The expression of the T-type calcium channel protein Cav3.2 was increased in the uterine smooth muscle of pregnant rats [21]; we further found that the Cav3.2 was overexpressed in the myometrial smooth muscle of infected preterm mice [53] and the disturbance of the Cav3.2 pathway attenuated absolute contraction of cells [54]. Thus, we aimed to define whether miR-25-3p participates in PTL by regulating HMSM contraction in vitro. However, to our surprise, the miR-25-3p level in HMSMs did not change by the stimulation with LPS. Considering that, the placenta is the closest tissue to the uterus during pregnancy; thus, we further detected the miR-25-3p in trophoblasts that were exposed to LPS. The decreased miR-25-3p in these trophoblasts suggests that the placental trophoblast may be more sensitive to external stimuli.

**Figure 8:**
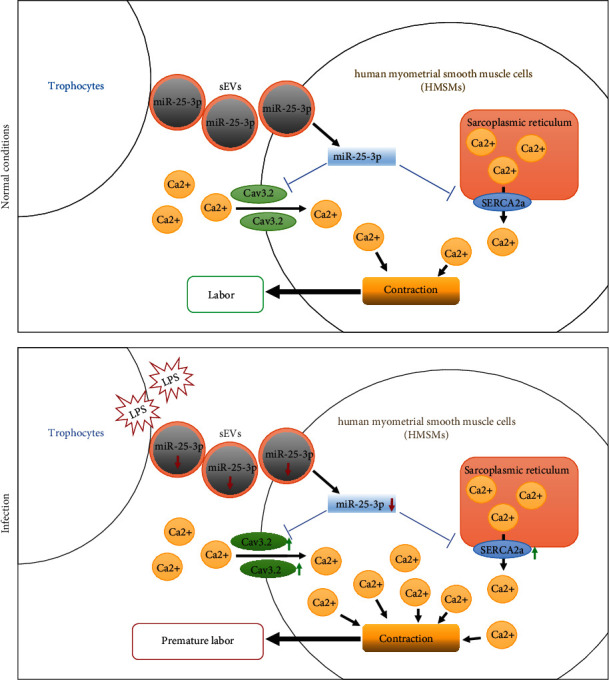
Proposed mechanism by which the exosomal miR-25-3p transmitted by the trophoblasts induced premature labor under infection conditions.

It has been proven that the placenta can trigger functional alterations in the maternal uterus by the secretion of sEVs [7]. The composition of the trophoblast sEVs is changed in different complications during pregnancy, which was attributed to hyperglycemia or hypoxia microenvironment [55–57]. Therefore, we speculate that bacterial infection may also affect the composition of trophoblast sEVs and thus regulate the contraction of the uterine myometrium. Our data showed that the sEVs derived from trophoblast cells could package miR-25-3p to the HMSMs; meanwhile, the expression of miR-25-3p was reduced in the sEVs from the trophoblasts that were pretreated with LPS. These sEVs further upregulated the expression of SERCA2a and Cav3.2 along with the activated Ca^2+^ release and Ca^2+^ entry in HMSMs. The above changes were all rescued by the forced upregulation of miR-25-3p. These results pumped us to speculate that under normal circumstances, the placenta transports a certain level of miR-25-3p to the myometrium through sEVs and maintains the calcium homeostasis of the myometrium. However, the infection caused a reduction of exosomal miR-25-3p that distorted the SERCA2a and Cav3.2 level in HMSMs, which thus caused Ca^2+^ oscillation and led to uterine contraction (Figure 8). The inhibition of miR-25-3p in the HMSMs reversed the responses induced by the sEVs derived from trophoblasts (which were transfected with miR-25-3p agomir and treated with LPS) emphasizing the essential role of miR-25-3p in these processes.

## 5. Conclusions

In conclusion, the present study described the phenomenon that trophoblasts affect the HMSM contraction via exosome-mediated exporting of miR-25-3p. The data also demonstrated that miR-25-3p could modulate the Ca^2+^ oscillation in HMSMs by the regulation of its targets Cav3.2 and SERCA2a. The current findings extend our knowledge to explain placental-maternal communication during PTL.

## Data Availability

The original contributions presented in the study are included in the article/Supplementary Materials. Further inquiries can be directed to the corresponding author.

## Supplementary Materials

Supplementary Materials Supplementary Table 1: the primer sequences used in the real-time PCR.

## References

[1] Vogel J. P., Chawanpaiboon S., Moller A. B., Watananirun K., Bonet M., Lumbiganon P. (2018). The global epidemiology of preterm birth. *Best Practice & Research. Clinical Obstetrics & Gynaecology*.

[2] Blencowe H., Cousens S., Oestergaard M. Z. (2012). National, regional, and worldwide estimates of preterm birth rates in the year 2010 with time trends since 1990 for selected countries: a systematic analysis and implications. *Lancet*.

[3] Platt M. J. (2014). Outcomes in preterm infants. *Public Health*.

[4] Ahumada-Barrios M. E., Alvarado G. F. (2016). Risk factors for premature birth in a hospital. *Revista Latino-Americana de Enfermagem*.

[5] Goldenberg R. L., Hauth J. C., Andrews W. W. (2000). Intrauterine infection and preterm delivery. *The New England Journal of Medicine*.

[6] Robertson S. A., Hutchinson M. R., Rice K. C. (2020). Targeting Toll-like receptor-4 to tackle preterm birth and fetal inflammatory injury. *Clinical & translational immunology*.

[7] Menon R., Mesiano S., Taylor R. N. (2017). Programmed fetal membrane senescence and exosome-mediated signaling: a mechanism associated with timing of human parturition. *Front Endocrinol (Lausanne)*.

[8] Mitchell M. D., Peiris H. N., Kobayashi M. (2015). Placental exosomes in normal and complicated pregnancy. *American Journal of Obstetrics and Gynecology*.

[9] Sarker S., Scholz-Romero K., Perez A. (2014). Placenta-derived exosomes continuously increase in maternal circulation over the first trimester of pregnancy. *Journal of Translational Medicine*.

[10] Luo S. S., Ishibashi O., Ishikawa G. (2009). Human villous trophoblasts express and secrete placenta-specific microRNAs into maternal circulation via exosomes. *Biology of Reproduction*.

[11] Menon R., Debnath C., Lai A. (2019). Circulating exosomal miRNA profile during term and preterm birth pregnancies: a longitudinal study. *Endocrinology*.

[12] Fallen S., Baxter D., Wu X. (2018). Extracellular vesicle RNAs reflect placenta dysfunction and are a biomarker source for preterm labour. *Journal of Cellular and Molecular Medicine*.

[13] Williams K. C., Renthal N. E., Gerard R. D., Mendelson C. R. (2012). The microRNA (miR)-199a/214 cluster mediates opposing effects of progesterone and estrogen on uterine contractility during pregnancy and labor. *Molecular Endocrinology*.

[14] Wray S., Jones K., Kupittayanant S. (2003). Calcium signaling and uterine contractility. *Journal of the Society for Gynecologic Investigation*.

[15] Noble D., Borysova L., Wray S., Burdyga T. (2014). Store-operated Ca^2+^ entry and depolarization explain the anomalous behaviour of myometrial SR: effects of SERCA inhibition on electrical activity, Ca^2+^ and force. *Cell Calcium*.

[16] Noble K., Matthew A., Burdyga T., Wray S. (2009). A review of recent insights into the role of the sarcoplasmic reticulum and Ca entry in uterine smooth muscle. *European Journal of Obstetrics, Gynecology, and Reproductive Biology*.

[17] Banciu A., Banciu D. D., Mustaciosu C. C. (2018). Beta-estradiol regulates voltage-gated calcium channels and estrogen receptors in telocytes from human myometrium. *International Journal of Molecular Sciences*.

[18] Aggarwal A., Bagga R., Girish B., Kalra J., Kumar P. (2018). Effect of maintenance tocolysis with nifedipine in established preterm labour on pregnancy prolongation and neonatal outcome. *Journal of obstetrics and gynaecology : the journal of the Institute of Obstetrics and Gynaecology*.

[19] Maher M. A., Sayyed T. M., El-Khadry S. W. (2019). Retracted: nifedipine alone or combined with sildenafil citrate for management of threatened preterm labour: a randomised trial. *BJOG : An International Journal of Obstetrics and Gynaecology*.

[20] Lee S. E., Ahn D. S., Lee Y. H. (2009). Role of T-type Ca channels in the spontaneous phasic contraction of pregnant rat uterine smooth muscle. *The Korean journal of physiology & pharmacology : official journal of the Korean Physiological Society and the Korean Society of Pharmacology*.

[21] Senadheera S., Bertrand P. P., Grayson T. H. (2013). Enhanced contractility in pregnancy is associated with augmented TRPC3, L-type, and T-type voltage-dependent calcium channel function in rat uterine radial artery. *American Journal of Physiology-Regulatory, Integrative and Comparative Physiology*.

[22] Young R. C., Zhang P. (2005). Inhibition of in vitro contractions of human myometrium by mibefradil, a T-type calcium channel blocker: support for a model using excitation-contraction coupling, and autocrine and paracrine signaling mechanisms. *Journal of the Society for Gynecologic Investigation*.

[23] Jing C., Dongming Z., Hong C., Quan N., Sishi L., Caixia L. (2018). TRPC3 overexpression promotes the progression of inflammation-induced preterm labor and inhibits T cell activation. *Cellular Physiology and Biochemistry : International Journal of Experimental Cellular Physiology, Biochemistry, and Pharmacology*.

[24] Wahlquist C., Jeong D., Rojas-Munoz A. (2014). Inhibition of _miR-25_ improves cardiac contractility in the failing heart. *Nature*.

[25] Jeong D., Yoo J., Lee P. (2021). miR-25 tough decoy enhances cardiac function in heart failure. *Molecular Therapy*.

[26] Hong Z., Chen K. H., DasGupta A. (2017). MicroRNA-138 and microRNA-25 down-regulate mitochondrial calcium uniporter, causing the pulmonary arterial hypertension cancer phenotype. *American journal of respiratory and critical care medicine*.

[27] Nold C., Stone J., O'Hara K. (2019). Block of granulocyte-macrophage colony-stimulating factor prevents inflammation-induced preterm birth in a mouse model for parturition. *Reproductive Sciences*.

[28] Deng W., Cha J., Yuan J. (2016). p53 coordinates decidual sestrin 2/AMPK/mTORC1 signaling to govern parturition timing. *The Journal of Clinical Investigation*.

[29] Loudon J. A., Sooranna S. R., Bennett P. R., Johnson M. R. (2004). Mechanical stretch of human uterine smooth muscle cells increases IL-8 mRNA expression and peptide synthesis. *Molecular Human Reproduction*.

[30] Friebe-Hoffmann U., Chiao J. P., Rauk P. N. (2001). Effect of IL-1beta and IL-6 on oxytocin secretion in human uterine smooth muscle cells. *American Journal of Reproductive Immunology*.

[31] Salomon C., Yee S., Scholz-Romero K. (2014). Extravillous trophoblast cells-derived exosomes promote vascular smooth muscle cell migration. *Frontiers in Pharmacology*.

[32] Salomon C., Guanzon D., Scholz-Romero K. (2017). Placental exosomes as early biomarker of preeclampsia: potential role of exosomal microRNAs across gestation. *The Journal of Clinical Endocrinology and Metabolism*.

[33] Ying W., Riopel M., Bandyopadhyay G. (2021). Adipose tissue macrophage-derived exosomal miRNAs can modulate in vivo and in vitro insulin sensitivity. *Cell*.

[34] Dalrymple A., Mahn K., Poston L., Songu-Mize E., Tribe R. M. (2007). Mechanical stretch regulates TRPC expression and calcium entry in human myometrial smooth muscle cells. *Molecular Human Reproduction*.

[35] Hashimoto K., Kajitani N., Miyamoto Y., Matsumoto K. I. (2018). Wound healing-related properties detected in an experimental model with a collagen gel contraction assay are affected in the absence of tenascin-X. *Experimental Cell Research*.

[36] Sussan T. E., Sudini K., Talbot C. C. (2017). Nrf2 regulates gene-environment interactions in an animal model of intrauterine inflammation: implications for preterm birth and prematurity. *Scientific Reports*.

[37] Rosen E. M., van't Erve T. J., Boss J. (2021). Urinary oxidative stress biomarkers and accelerated time to spontaneous delivery. *Free Radical Biology and Medicine*.

[38] Thota C., Farmer T., Garfield R. E., Menon R., Al-Hendy A. (2013). Vitamin D elicits anti-inflammatory response, inhibits contractile-associated proteins, and modulates Toll-like receptors in human myometrial cells. *Reproductive Sciences*.

[39] Mani A., Hotra J. W., Blackwell S. C., Goetzl L., Refuerzo J. S. (2020). Mesenchymal stem cells attenuate lipopolysaccharide-induced inflammatory response in human uterine smooth muscle cells. *AJP Rep*.

[40] Zhang L., Wang L., Jiang J., Zheng D., Liu S., Liu C. (2015). Lipopolysaccharides upregulate calcium concentration in mouse uterine smooth muscle cells through the T-type calcium channels. *International Journal of Molecular Medicine*.

[41] Kambe S., Yoshitake H., Yuge K. (2014). Human exosomal placenta-associated miR-517a-3p modulates the expression of PRKG1 mRNA in Jurkat cells. *Biology of Reproduction*.

[42] Tribe R. M., Moriarty P., Dalrymple A., Hassoni A. A., Poston L. (2003). Interleukin-1beta induces calcium transients and enhances basal and store operated calcium entry in human myometrial smooth muscle. *Biology of Reproduction*.

[43] Africa C. W. (2011). Oral colonization of Gram-negative anaerobes as a risk factor for preterm delivery. *Virulence*.

[44] Vallely L. M., Egli-Gany D., Pomat W. (2021). Adverse pregnancy and neonatal outcomes associated with Neisseria gonorrhoeae, Mycoplasma genitalium, M. hominis, Ureaplasma urealyticum and U. parvum: a systematic review and meta-analysis protocol. *BMJ Open*.

[45] Thaxton J. E., Nevers T. A., Sharma S. (2010). TLR-mediated preterm birth in response to pathogenic agents. *Infectious diseases in obstetrics and gynecology*.

[46] Sun X., Deng W., Li Y. (2016). Sustained endocannabinoid signaling compromises decidual function and promotes inflammation-induced preterm birth∗. *The Journal of Biological Chemistry*.

[47] Weber D., Stuetz W., Bernhard W. (2014). Oxidative stress markers and micronutrients in maternal and cord blood in relation to neonatal outcome. *European Journal of Clinical Nutrition*.

[48] Negi R., Pande D., Kumar A., Khanna R. S., Khanna H. D. (2012). Evaluation of biomarkers of oxidative stress and antioxidant capacity in the cord blood of preterm low birth weight neonates. *The Journal of Maternal-Fetal & Neonatal Medicine*.

[49] Cipierre C., Hays S., Maucort-Boulch D., Steghens J. P., Picaud J. C. (2013). Adduct of malondialdehyde to hemoglobin: a new marker of oxidative stress that is associated with significant morbidity in preterm infants. *Oxidative Medicine and Cellular Longevity*.

[50] Li R., Wen Y., Wu B. (2020). MicroRNA-25-3p suppresses epileptiform discharges through inhibiting oxidative stress and apoptosis via targeting OXSR1 in neurons. *Biochemical and Biophysical Research Communications*.

[51] Li Z., Ali Shah S. W., Zhou Q., Yin X., Teng X. (2021). First-principles Investigations on a Two-dimensional S3N2/Black Phosphorene van der Waals Heterostructure: Mechanical, Carrier Transport and Thermoelectric Anisotropy. *Environmental Pollution*.

[52] Khan I., Tabb T., Garfield R. E. (1993). Expression of the internal calcium pump in pregnant rat uterus. *Cell Calcium*.

[53] Chen J., Zheng D., Cui H., Liu S., Zhang L., Liu C. (2018). Roles and mechanisms of TRPC3 and the PLC*γ*/PKC/CPI-17 signaling pathway in regulating parturition. *Molecular Medicine Reports*.

[54] Wan J., Yamamura A., Zimnicka A. M. (2013). Chronic hypoxia selectively enhances L- and T-type voltage-dependent Ca2+ channel activity in pulmonary artery by upregulating Cav1.2 and Cav3.2. *American Journal of Physiology-Lung Cellular and Molecular Physiology*.

[55] Baig S., Kothandaraman N., Manikandan J. (2014). Proteomic analysis of human placental syncytiotrophoblast microvesicles in preeclampsia. *Clinical Proteomics*.

[56] Guller S., Tang Z., Ma Y. Y., Di Santo S., Sager R., Schneider H. (2011). Protein composition of microparticles shed from human placenta during placental perfusion: potential role in angiogenesis and fibrinolysis in preeclampsia. *Placenta*.

[57] Baig S., Lim J. Y., Fernandis A. Z. (2013). Lipidomic analysis of human placental syncytiotrophoblast microvesicles in adverse pregnancy outcomes. *Placenta*.

