# Supplementary material for: Trophoblasts Modulate the Ca2+ Oscillation and Contraction of Myometrial Smooth Muscle Cells by Small Extracellular Vesicle- (sEV-) Mediated Exporting of miR-25-3p during Premature Labor
Source: Oxid Med Cell Longev. 2021 Aug 7;2021:8140667. doi: 10.1155/2021/8140667 (PMC8369173; doi:10.1155/2021/8140667)
Supplement: Supplementary Materials — Supplementary Table 1: the primer sequences used in the real-time PCR. [file 8140667.f1.docx]

**Supplementary Table 1. The primer sequences used in the real-time PCR**

| **Primers** | | **Sequence (5’-3’)** |
| --- | --- | --- |
| miR-25-3p | RT primer | GTTGGCTCTGGTGCAGGGTCCGAGGTATTCGCACCAGAGCCAACTCAGAC |
|  | PCR primer | Forward: CATTGCACTTGTCTCGGTCTGA |
|  |  | Reverse: GTGCAGGGTCCGAGGTATTC |
| ATP2A2 | PCR primer | Forward: GACCCTTGGTTGTACTTCTG |
|  |  | Reverse: CACTGGCTTATCATCCTTTT |
| CACNA1H | PCR primer | Forward: AGCCGTTGGCGTAAGAA |
|  |  | Reverse: GCTGAAGTGGTAATGGTGGT |
